# Evaluation of radiation sensitivity and mating performance of *Glossina brevipalpis* males

**DOI:** 10.1371/journal.pntd.0005473

**Published:** 2017-03-17

**Authors:** Chantel J. de Beer, Percy Moyaba, Solomon N. B. Boikanyo, Daphney Majatladi, Hanano Yamada, Gert J. Venter, Marc J. B. Vreysen

**Affiliations:** 1 Parasites, Vectors & Vector-borne Diseases, Agricultural Research Council-Onderstepoort Veterinary Institute (ARC-OVI), Onderstepoort, South Africa; 2 Department of Zoology and Entomology, University of the Free State, Bloemfontein, South Africa; 3 Insect Pest Control Laboratory, Joint FAO/IAEA Division of Nuclear Techniques in Food and Agriculture, Seibersdorf, Austria; 4 Department of Veterinary Tropical Diseases, University of Pretoria, Onderstepoort, South Africa; Institut de recherche pour le developpement, FRANCE

## Abstract

**Background:**

Area-wide integrated pest management strategies that include a sterile insect technique component have been successfully used to eradicate tsetse fly populations in the past. To ensure the success of the sterile insect technique, the released males must be adequately sterile and be able to compete with their native counterparts in the wild.

**Methodology/Principal findings:**

In the present study the radiation sensitivity of colonised *Glossina brevipalpis* Newstead (Diptera; Glossinidae) males, treated either as adults or pupae, was assessed. The mating performance of the irradiated *G*. *brevipalpis* males was assessed in walk-in field cages. *Glossina brevipalpis* adults and pupae were highly sensitive to irradiation, and a dose of 40 Gy and 80 Gy induced 93% and 99% sterility respectively in untreated females that mated with males irradiated as adults. When 37 to 41 day old pupae were exposed to a dose of 40 Gy, more than 97% sterility was induced in untreated females that mated with males derived from irradiated pupae. Males treated as adults with a dose up to 80 Gy were able to compete successfully with untreated fertile males for untreated females in walk-in field cages.

**Conclusions/Significance:**

The data emanating from this field cage study indicates that, sterile male flies derived from the colony of *G*. *brevipalpis* maintained at the Agricultural Research Council-Onderstepoort Veterinary Institute in South Africa are potential good candidates for a campaign that includes a sterile insect technique component. This would need to be confirmed by open field studies.

## Author summary

The radiation sensitivity and mating performance of colonised *G*. *brevipalis* adult and pupae were evaluated. It was showed that *G*. *brevipalpis* males were highly sensitive to irradiation and could be sterilised at a much lower dose, between 40 Gy and 80 Gy, than some other tsetse fly species. As a result, males sterilised with a dose of up to 80 Gy performed similar as that of un-irradiated males for untreated females in walk-in field ages. The data from the study indicate that, under the experimental field cage conditions, the *G*. *brevipalpis* males derived from the colony at ARC-OVI should be well suited for use in AW-IPM programmes that have a SIT component.

## Introduction

Tsetse flies (Diptera; Glossinidae), the cyclical vectors of trypanosome parasites that cause human African trypanosomosis (HAT) and African animal trypanosomosis (AAT), infest 10 million km² of sub-Saharan Africa [1–3]. In South Africa, *Glossina brevipalpis* Newstead and *Glossina austeni* Newstead are the vectors of *Trypanosoma congolense* and *Trypanosoma vivax* [4–7] that cause AAT in an area of about 16 000 km^2^ in the north eastern parts of the KwaZulu-Natal Province [8,9]. Because of the limited options available for controlling the parasite, i.e. no efficient vaccines and increased resistance to the commonly used trypanocidal drugs [1,10], vector control remains an effective and economical option for managing AAT in KwaZulu-Natal.

The use of sterile males to control field populations of insect pests, as conceptualised by E.F. Knipling in the 1940s [11–13], has been applied successfully to control tsetse fly populations. In the 1970 and 1980’s, the sterile insect technique (SIT) was used in feasibility studies against populations of *Glossina morsitans morsitans* Westwood in Zimbabwe and Tanzania, *Glossina tachinoides* Westwood in Chad and *Glossina palpalis gambiensis* Vanderplank in Burkina Faso [13–19]. The first eradication campaign that integrated the use of radiation-sterilized adult male tsetse with suppression methods such as insecticide-impregnated targets, was implemented against *Glossina morsitans submorsitans* Newstead, *G*. *p*. *gambiensis* and *G*. *tachinoides* in Burkina Faso in the 1980’s [20]. A similar approach was used around the same period to target a population of *Glossina palpalis palpalis* (Robineau-Desvoidy) in an agro-pastoral area of Lafia in Nigeria [21]. All targeted populations of the four species were eradicated from the controlled zones. These programmes, although successful, were however not conducted area-wide, and their pest free status was lost due to reinvasion from neighbouring areas [13].

The most successful AW-IPM programme with a SIT component against a tsetse fly population to date was implemented on Unguja Island of Zanzibar, Tanzania in the 1990’s [22]. Suppression of *G*. *austeni* populations with insecticide-treated screens and cattle started in 1988 and from August 1994 up to December 1997, 8.5 million sterile flies were released on the island [22]. The last wild *G*. *austeni* was trapped in September 1996 and to date, Unguja Island is still free of tsetse flies and trypanosomosis. Initial results of an AW-IPM campaign with a SIT component currently underway against a *G*. *p*. *gambiensis* population in the Niayes of Senegal, seems likewise promising [23–25].

The SIT entails the use of radiation to sterilise males of the target species reared in a mass-rearing facility [11,26], followed by the sequential and area-wide release of these males in sufficient numbers to outcompete their wild counterparts [13,27]. The mating of sterile males with wild fertile females results in no progeny, which leads to reduction in the size of the targeted population and, in some cases, to local eradication [27]. Because the released sterile males must be able to outcompete the local wild males in mating with wild females, the biological quality and sexual competitiveness of the sterile males are of utmost importance [28,29]. Releasing low quality sterile males will necessitate higher release rates and might prolong the duration of the programme, which will require more funding and the potential for failure will be higher [28].

Ionizing radiation can potentially influence the mating competiveness of the released males. The effect of gamma radiation on the reproduction and competitiveness of several tsetse species was investigated in the past [27–34]. Radiation doses ranging from 50 Gy for *G*. *brevipalpis* up to 170 Gy for *G*. *tachinoides* induced acceptable or complete sterility in these two species [30,31]. Doses of 110 Gy to 120 Gy induced sterility in *G*. *austeni* [32], *G*. *tachinoides* [33], *G*. *p*. *palpalis* [34], *Glossina pallidipes* Austen [35] and *G*. *p*. *gambiensis* [36]. It was showed that a dose of 120 Gy did not affect mating competitiveness of *G*. *pallidipes* males in walk-in field cages and keeping the irradiated pupae for 24–72 h at a low temperature of 15°C even increased the competitiveness of the emerged males [37].

The successful and sustained removal of *G*. *austeni* from the Island of Unguja [22] demonstrated the feasibility of using the SIT to eradicate a population of this species. However, the SIT has never been used or evaluated against the second tsetse fly species present in South Africa i.e. *G*. *brevipalpis*, and no data is available on the mating performance of irradiated flies.

The distribution of *G*. *brevipalpis* extends from Ethiopia in the north to KwaZulu-Natal, South Africa in the south and is present in Somalia, Uganda, Kenya, Rwanda, Burundi, Tanzania, Malawi, Zambia, Zimbabwe and Mozambique [38]. Although this species belongs to the fusca group of tsetse flies, considered of less or no epidemiological importance for livestock, it was recently shown that the role of this species in transmitting AAT in South Africa should not be underestimated [9]. It has become evident that removing only *G*. *austeni* from KwaZulu-Natal would not solve the AAT problem and any potential AW-IPM campaign with a SIT component should include *G*. *brevipalpis* [9].

With the exception of a study in the 1990s, that showed that a radiation dose of 50 Gy administered to adult *G*. *brevipalpis* males induced 95% sterility [31] very little is known on the biology of irradiated *G*. *brevipalpis* flies. We therefore assessed the radiation sensitivity of adult *G*. *brevipalpis* males when treated as pupae and adults, as well as their mating performance in walk-in field cages.

## Materials and methods

### Ethical statement

Materials used in the study posed no health risk to researchers and no vertebrate animals were involved. Permission to do research in terms of Section 20 of the animal diseases act South Africa (ACT no. 35 of 1984) has been granted for tsetse fly colony maintenance, Ref 12/11/1/1.

### Colony tsetse flies

*Glossina brevipalpis* adults and pupae were obtained from a laboratory colony housed at the Agricultural Research Council-Onderstepoort Veterinary Institute (ARC-OVI), Pretoria, South Africa. This colony was established in 2002 using seed material from the FAO/IAEA Insect Pest Control Laboratory in Austria. The colony flies were maintained on abattoir collected defibrinated bovine blood using an artificial *in vitro* membrane feeding system under standardised environmental conditions (23–24°C, 75–80% RH and subdued/indirect illumination, 12h light/12h dark) [39,40].

### Radiation sensitivity

To determine the radiation sensitivity of *G*. *brevipalpis*, adult males and pupae (both males and females) were irradiated with either 40 Gy, 80 Gy, 100 Gy, 120 Gy or 140 Gy using a ^137^Cs source (Gammacell 40 S/N50) at a dose rate of 0.69 Gy/min in air. Adult males were irradiated four days after emergence. To synchronise adult emergence from irradiated pupae, pupae were collected from the colonies in 24-hour increments. Pupae were irradiated on day 41 (group 1), 39 (group 2) or 37 (group 3) post-larviposition (PL), i.e. three-, five- and seven days before expected emergence. Only the males from the irradiated pupae were used.

To determine the effect of radiation on reproduction, 15 six-day-old treated (un-irradiated for the control or irradiated as adults or pupae) males were mated with 30 three-day-old un-irradiated virgin females at a 1:2 male:female ratio. Males and females were allowed to mate for four days in standard colony holding cages (Ø 20 cm). Thereafter they were kept separately under standard colony conditions [39,40]. Each treatment dose was replicated three to four times.

Male and female survival was monitored daily and pupae production recorded. Fecundity was defined as the number of pupae produced per mature female day [31,32]. Mature female days were calculated for each treatment by adding the number of flies alive each day, from day 18 (first larviposition day) after emergence until the end of the experiment on day 60 [32].

All pupae produced were mechanically sorted into one of five class sizes (A (3.5 mm) to E (4.3 mm)) according to the standards used by the FAO/IAEA Insect Pest Control Laboratory. The pupal size classes had the following associated weight (mg): A (<56), B (56– <68), C (68– <76), D (76– <84), and E (≥84) and adult emergence was recorded [39,40]. Aborted eggs and immature larval stages were monitored daily. After 60 days, all surviving females were dissected to determine their reproductive status, insemination rate and spermathecal fill [39,40]. The spermathecae were removed and their fill microscopically scored as empty (0), quarter full (0.25), half (0.5), three quarters full (0.75) or full (1) [41]. Male mortality was monitored until all the males had died.

### Male mating performance

Comparative assessment of the mating performance of gamma sterilised *G*. *brevipalpis* males was conducted during February 2013 in walk-in field cages [37,42] deployed in a small forest under near-natural conditions at the ARC-OVI [43]. The cylindrical walk-in field cages (Ø 2.9 m x 2.0 m) were made of cream polyester netting with a flat floor and ceiling. Black nylon bands, connecting the ceiling and floor with the sides, encircled the top and bottom of the cage. A 1.5 m potted *Schotia brachypetala* was placed in the middle of the cage. Throughout the experiment, temperature and relative humidity were recorded every 10 minutes using a DS1923-F5# Hygrochron iButton data logger (Fairbridge technologies, South Africa). Light intensity at the top and bottom of the cage and at tree level was recorded every 15 minutes using a Major Tech MT940 light meter.

It was previously determined that the optimal mating age for *G*. *brevipalpis* males was nine days [43]. Therefore, 30 nine-day-old males irradiated four days after emergence with either 40 Gy or 80 Gy and a group of 30 nine-day old un-irradiated males competed for 30 three-day-old virgin females, giving a male to female ratio of 2:1 in each field cage at the commencement of the experiment. The male groups were distinguished by a dot of different coloured polymer paint placed on the notum 24 hours before being released in the field cage [37].

Experiments were conducted from 12:00 h to 15:00 h. Females were released 5 minutes before the males in the middle of the cage, and the time of the first mating recorded to determine mating latency The observer remained inside the cage for the 3-hour duration of the experiment and movements were kept to a minimum. The mating pairs were collected individually into small vials, and duration of the mating recorded. Although no direct adverse effect on mating behaviour was observed when the pairs were collected, its potential influence on mating behaviour cannot be ruled out. To minimise this effect mating pairs were collected in the same way in all experiments. They were not replaced.

Females that did not mate in the field cage by the end on the 3-hour duration were collected, immobilised at -5°C and dissected to confirm virginity. The mated females were transferred from the small vials to individual holding cages and kept under standard colony conditions. The females were kept for 60 days and monitored daily for survival and production similar to that of the radiation sensitivity experimental flies, except that the pupae produced were not classed by size. Females were dissected after 60 days to determine reproductive status.

Mating performance was assessed by the following mating indices: the propensity of mating (PM), relative mating index (RMI) and relative mating performance (RMP). Propensity of mating (PM) was defined as the overall proportion of released females that had mated. Relative mating index (RMI) was defined as the number of pairs of one treatment group as a proportion of the total number of matings [37]. Relative mating performance (RMP) was defined as the difference between the number of matings of two treatments of males as a proportion of the total number of matings [37]. In addition, the mating latency time, mating duration, insemination rate and the spermathecal fill of each mated female were determined.

### Statistical analysis

Data were analysed using the statistical software GraphPad Instat [44] and R [45]. Proportional differences in adult emergence rates were determined with Chi-square (*χ*^2^) analysis with the Yate’s continuity correction. The P value was two-sided. Linear regression analysis was used to assess effect of radiation dose on fecundity. The effect of time of irradiation and dose on male survival was analysed with multivariate linear regression model:

Estimated survival=β0+β1⋅age(days)+β2⋅dose(Gy)

A one-way analysis of variance (ANOVA) was used to differentiate between the relative mating index, average mating latency, mating duration and spermathecal fill. If the data were normally distributed standard (parametric) methods were used and the Tukey’s test was applied otherwise a nonparametric Kruskal-Wallis test was used. Tests were done at the 5% significance level.

## Results

### Radiation sensitivity

#### Adult emergence rate

Female emergence started on day 37 to 41 PL and peaked on day 39 to 42 PL. Male emergence started on day 41 PL for group 1 and on day 43 PL for group 3 and peaked on day 44 PL for group 1 and day 45 PL for group 3. The last males emerged on day 46 and 47 PL for groups 1 and 3, respectively. Total emergence time ranged from 4 to 7 days in un-irradiated flies. In irradiated pupa, this period was somewhat prolonged and ranged from 4 (group 1; 40 Gy) to 10 days (group 2; 140 Gy). The differences in the emergence period between pupae irradiated 41, 39 or 37 PL was within the normal variation as seen in the non-irradiated pupae control of each group. Although the irradiation did not affect the day emergence started, it extended the total period of emergence.

Adult emergence rate from pupae irradiated with the five doses was compared with that of the untreated control group (Table 1). Emergence from group 1 pupae irradiated with 100, 120 or 140 Gy was significantly (P < 0.01 in all cases) lower than that of the untreated control group (95.2%) (Table 1). For group 2 pupae adult emergence from pupae irradiated with 40 Gy (P = 0.03), 80 Gy (P = 0.03) and 120 Gy (P < 0.01) was significantly lower than that of the control (77.3%) (Table 1). Adult emergence from group 3 pupae irradiated with 140 Gy was significantly higher (P = 0.04) than that of the untreated control (69.6%) (Table 1). On average the male to female ratio of adults that emerged from any of the irradiated groups did not differ significantly from that of the untreated control group (1:1) (Table 1).

**Table 1:** Emergence rate of adult *Glossina brevipalpis* irradiated as pupae with different doses and on different days Post Larviposition (PL).

Radiation dose (Gy)	No. pupae	Males (%)	Females (%)	Total (%)	Male: Female ratio
**Grp 1: Pupae irradiated 41 days PL**					
Control	167	88 (55.4)	71 (44.7)	159 (95.2)	1: 0.8
40	167	88 (54.7)	73 (45.3)	161 (96.4)	1: 0.8
80	167	74 (49.7)	75 (50.3)	149 (89.2)	1: 1
100	167	63 (48.5)	67 (51.5)	130 (77.8)*	1: 1.1
120	167	47 (38.5)	75 (61.5)	122 (73.1)*	1: 1.6
140	167	72 (57.6)	53 (42.4)	125 (74.9)*	1: 0.7
**Grp 2: Pupae irradiated 39 days PL**					
Control	132	65 (63.7)	37 (36.3)	102 (77.3)	1: 0.6
40	132	57 (67.1)	28 (32.9)	85 (64.4)*	1: 0.5
80	132	59 (69.4)	26 (30.6)	85 (64.4)*	1: 0.5
100	132	59 (60.8)	38 (39.2)	97 (73.5)	1: 0.6
120	132	32 (43.8)	41 (56.2)	73 (55.3)*	1: 1.3
140	132	50 (54.4)	42 (45.7)	92 (69.7)	1: 0.8
**Grp 3: Pupae irradiated 37 days PL**					
Control	112	34 (43.6)	44 (56.4)	78 (69.6)	1: 1.3
40	112	38 (52.8)	34 (47.2)	72 (64.3)	1: 0.9
80	112	44 (55.0)	36 (45.0)	80 (71.4)	1: 0.8
100	112	44 (54.3)	37 (45.7)	81 (72.3)	1: 0.8
120	112	47 (57.3)	35 (42.7)	82 (73.2)	1: 0.7
140	112	55 (59.8)	37 (40.2)	92 (82.1)*	1: 0.7

*Emergence rate statistical significant different from the control group at the 5% level.

#### Fecundity of females mated with males irradiated as adults or pupae

Of 2820 females (30 for each treatment replicate), 2668 survived up to day 18 after emergence. The survival of mature females in all individual experiments exceeded 95.9% on day 18, except for group 3 pupae (irradiated day 37 PL) where it was still relative high at 88.8%.

Fecundity (number of pupae produced per mature female) was dose dependent and decreased as radiation doses increased (Table 2). A negative linear regression was found between fecundity of untreated females that mated with males irradiated as adults at the five radiation levels (*r*^*2*^ = 0.62, P < 0.01), or males that had been irradiated as pupae in all of the pupal groups (*r*^*2*^ = 0.57, P = 0.01). Untreated females that had mated with adult males exposed to 40 and 80 Gy produced 16 and 2 pupae, respectively which represented a production of 6.7% and 0.8% respectively, relative to that of the untreated controls (n = 240). Pupal production (Table 2) relative to the untreated control group for the females mated with males irradiated with 40 Gy as pupae was 1.4%, 1.9%, and 2.9% for group 1, 2 and 3, respectively. A dose of 40 and 80 Gy was sufficient to induce 93% and 99% sterility, respectively in untreated females when the males had been irradiated as adults. Induced sterility was 97% or higher when the males had been irradiated with 40 Gy for the different groups of pupae.

**Table 2:** Production parameters of *Glossina brevipalpis* females mated with males irradiated with different doses at different days Post Larviposition (PL).

					Pupal size classes (mg)						
Radiation dose (Gy)	Replications	No. of mature females (day 18)	No. of aborted eggs	No. pupae produced	A (%)	B (%)	C (%)	D (%)	E (%)	Fecundity^#^	% Emergence/ % females
					<56	56-<68	68-<76	76-<84	>84		
**Irradiated male adults**											
Control	4	119	36	240	7 (2.9)	18 (7.5)	60 (25.0)	85 (35.4)	79 (29.2)	0.046	92.9 / 49.3
40	4	116	341	16	2 (12.5)	1 (6.3)	6 (37.5)	3 (18.8)	4 (25.0)	0.003	93.8 / 46.7
80	4	114	326	2	0	1 (50.0)	0	1 (50.0)	0	0	2 / 2 / 1 / 2
100	4	119	402	0	0	0	0	0	0	0	
120	4	112	287	0	0	0	0	0	0	0	
140	4	119	307	0	0	0	0	0	0	0	
**Grp 1: Pupae irradiated 41 days PL**											
Control	4	114	20	294	15 (6.2)	45 (18.7)	87 (36.1)	68 (28.2)	26 (10.8)	0.060	67.2 / 61.4
40	4	116	248	4	0	0	2 (100)	0	0	0.001	3 /4 /2 / 4
80	4	116	218	0	0	0	0	0	0	0	
100	4	109	187	1	1 (100)	0	0	0	0	<0.001	0 / 1 / 0 / 1
120	4	119	240	1	0	0	0	1 (100)	0	<0.001	0 / 1 / 0 / 1
140	3	88	152	0	0	0	0	0	0	0	
**Grp 2: Pupae irradiated 39 days PL**											
Control	4	115	13	269	25 (9.3)	59 (21.9)	92 (34.1)	83 (30.7)	11 (4.1)	0.057	86.3 / 58.2
40	4	115	230	5	1 (20)	2 (40)	1 (20)	1 (20)	0	0.001	80.0 / 25.0
80	4	118	252	0	0	0	0	0	0	0	
100	4	113	232	3	3 (100)	0	0	0	0	0.001	66.7 / 50.0
120	4	116	223	1	0	1 (100)	0	0	0	<0.001	0 / 1 / 0 / 1
140	4	117	221	1	0	0	0	0	0	<0.001	0 / 1 / 0 / 1
**Grp 3: Pupae irradiated 37 days PL**											
Control	4	116	10	343	24 (7.8)	64 (20.7)	109 (35.3)	90 (29.1)	22 (71.2)	0.058	91.8 / 52.9
40	4	106	181	10	1 (11.1)	3 (33.3)	6 (55.6)	0	0	0.023	30.0 / 66.7
80	4	111	176	0	0	0	0	0	0	<0.001	
100	4	109	174	0	0	0	0	0	0	0	
120	4	96	150	2	0	1 (50)	1 (50)	0	0	0.001	0 / 0 / 0 / 0
140	3	75	118	2	0	0	2 (100)	0	0	<0.001	0 / 0 / 0 / 0
**Adults from field cage**											
Fertile	4	74	14	81						0.058	91.5 / 44.7
Sterile (40 Gy)	4	65	52	10						0.010	100.0 / 25.0
Sterile (80 Gy)	4	53	37	2						0.006	75.0 / 25.0

^#^ Number of pupa produced per the mature female day

The number of aborted eggs during the 60-day trial was lower in females that mated with non-irradiated males than in those that mated with males of any of the experimental groups (Table 2). The majority of the pupae produced were in or above the pupal size class C (Table 2) for the control group as well as all treatment groups. The male to female ratio that emerged from pupae produced by females mated with irradiated males were similar and equally distributed (Table 2).

Dissections at the end of the experimental period of 60 days showed that the average insemination rate in females that mated with males irradiated as adults was higher than the insemination rate in females that mated with the control males (98.0% ± 3.55 versus 92.2%). This clearly showed that irradiation of adult males did not affect their ability to transfer sperm. Similar high insemination rates, independent of dose, were observed when the males were irradiated as pupae (Table 3).

**Table 3:** Reproductive status of *Glossina brevipalpis* females mated with irradiated males at different days Post Larviposition (PL) and radiation levels dissected after an experimental period of 60 days.

						Uterus				
		Spermathecal fill						Viable instar larvae		
Radiation dose (Gy)	Insemination %	0.25 (%)	0.5 (%)	0.75 (%)	1 (%)	Recently ovulated egg (%)	Empty due to abortion (%)	I (%)	II (%)	III (%)
**Irradiated male adults**										
Control	92.2	16 (13.8)	27 (23.3)	60 (51.7)	4 (3.5)	50 (44.2)	17 (15.0)	16 (14.2)	14 (12.4)	16 (14.2)
40	100.0	10 (8.9)	25 (22.1)	62 (54.9)	16 (14.2)	15 (13.3)	98 (86.7)	0	0	0
80	91.9	23 (20.7)	29 (26.1)	37 (33.3)	13 (11.7)	8 (7.2)	103 (92.8)	0	0	0
100	99.1	5 (4.3)	33 (28.5)	56 (48.3)	21 (18.1)	18 (15.5)	98 (84.5)	0	0	0
120	100.0	15 (14.0)	36 (33.6)	40 (37.4)	16 (15.0)	19 (17.8)	88 (82.2)	0	0	0
140	100.0	0	53 (48.2)	46 (41.8)	11 (10.0)	31 (28.2)	79 (71.8)	0	0	0
**Grp 1: Pupae irradiated 41 days PL**										
Control	94.6	13 (11.7)	35 (31.5)	57 (51.4)	0	65 (58.6)	23 (20.7)	7 (6.3)	9 (8.1)	7 (6.3)
40	92.7	18 (16.4)	36 (32.7)	48 (43.6)	0	31 (29.0)	76 (71.0)	0	0	0
80	87.6	16 (14.2)	46 (40.7)	33 (29.2)	4 (3.5)	18 (15.9)	95 (84.1)	0	0	0
100	95.3	28 (26.2)	32 (29.9)	41 (38.3)	1 (0.9)	21 (19.6)	86 (80.4)	0	0	0
120	97.4	27 (23.3)	35 (30.2)	51 (44.0)	0	38 (32.8)	78 (67.2)	0	0	0
140	89.9	18 (20.2)	35 (28.1)	35 (39.3)	2 (2.3)	10 (11.6)	76 (88.4)	0	0	0
**Grp 2: Pupae irradiated 39 days PL**										
Control	96.9	5 (5.2)	22 (22.9)	63 (65.6)	3 (3.1)	29 (31.2)	12 (12.9)	6(6.5)	15 (16.1)	31 (33.3)
40	95.9	14 (14.4)	33 (34.0)	45 (46.4)	1 (1.0)	26 (25.7)	75 (74.3)	0	0	0
80	94.3	12 (11.4)	41 (39.1)	45 (42.9)	1 (1.0)	23 (22.1)	81 (77.9)	0	0	0
100	94.3	18 (17.1)	18 (17.1)	60 (57.1)	3 (2.9)	20 (18.9)	86 (81.1)	0	0	0
120	97.2	12 (11.1)	34 (31.5)	58 (53.7)	1 (0.9)	28 (25.9)	79 (73.2)	1 (0.9)	0	0
140	96.4	16 (14.6)	44 (40.0)	46 (41.8)	0	22 (20.0)	88 (80.0)	0	0	0
**Grp 3: Pupae irradiated 37 day PL**										
Control	97.1	11 (10.5)	26 (24.8)	64 (61.0)	1 (1.0)	49 (47.1)	24 (23.1)	1 (1.0)	14 (13.5)	16 (15.3)
40	100.0	7 (7.9)	26 (29.2)	55 (61.8)	1 (1.1)	27 (30.3)	61 (68.5)	1 (1.2)	0	0
80	97.9	14 (14.1)	26 (26.3)	56 (56.6)	1 (1.0)	28 (28.3)	71 (71.7)	0	0	0
100	97.7	11 (12.8)	23 (26.7)	50 (58.1)	0	15 (17.9)	69 (82.1)	0	0	0
120	93.9	12 (14.5)	28 (33.7)	38 (45.8)	0	17 (20.5)	66 (79.5)	0	0	0
140	96.9	2 (3.1)	28 (43.8)	32 (50.0)	0	16(25.0)	48 (75.0)	0	0	0
**Adults from field cage**										
Fertile	100.0	7 (10.1)	26 (37.7)	36 (52.2)	0	27 (39.1)	17 (24.6)	5 (7.2)	6 (8.7)	14 (20.4)
Sterile (40 Gy)	100.0	6 (10.7)	32 (57.1)	18 (32.1)	0	19 (33.9)	35 (62.5)	0	0	2 (3.6)
Sterile (80 Gy)	98.0	3 (6.3)	19 (39.6)	25 (52.1)	0	8 (16.7)	40 (83.3)	0	0	0

It also showed that the reproductive status of females mated with untreated males was markedly different from that of females that mated with irradiated males (Table 3). The uteri of females that mated with males irradiated as adults were, either empty due to abortions or contained a recently ovulated egg or an egg in embryonic arrest. The same trend, irrespective of dose, was also observed for the pupal treatment groups (Table 3). In contrast, the uteri of most of the females that mated with un-irradiated males contained either a recently ovulated egg or a viable larva (Table 3).

#### Male survival

For the first two weeks after emergence, neither the age at time of irradiation (β1) nor the dose (β2) had any significant effect on male survival (Table 4). The irradiation of older pupa or adult flies had a positive effect on male survival and this was significant from 28 days post emergence (PE) (β1 = 0.027, P = 0.02), with an increase of 2.7% for every additional day in age at which irradiated. This positive effect further increases the older the insects become: for example, at 70 days, an increase of 1 day of the age of the pupae (at time of irradiation), will have a positive impact on survival rate (i.e. the survival rate will increase by 0.056 or 5.6%). The negative effects of irradiation do not become significant until day 56 post-emergence, where every increase in dose by 1 Gy impacts survival rate negatively by 0.15% (as seen by the constant β2).

**Table 4:** Effect of the developmental stage (age of pupa or adult) (β_1_) at time of and dose (β_2_) of irradiation on the survival of the male flies determined at 14 day intervals.

14 day intervals	Constant (β_0_)	Time of radiation (β_1_)	Dose (β_2_)	*r*^*2*^
	Mean (standard error)	Mean (standard error)	Mean (standard error)	
14	0,7409 (0,4049)	0,0015 (0,0099)	0,0002 (0,0006)	0,007
28	-0,3618 (0,4329)	0,0270 (0,0106) *	-0,0003 (0,0007)	0,242
42	-0,8658 (0,4030)	0,0382 (0,0098) ***	-0,0007 (0,0006)	0,439
56	-1,4311 (0,3790)	0,0511 (0,0093) ***	-0,0015 (0,0006) *	0,640
70	-1,6962 (0,326)	0,0565 (0,0081) ***	-0,0029 (0,0005) ***	0,795

*Statistical significant at the 5% level, * p<0.05,

*** p<0.001.

### Male mating performance

#### Environmental conditions and fly activity in the field cage

The field cage experiments were conducted in the afternoon from 12:00 h to15:00 h in February 2013. The ambient temperature ranged from 23.1°C to 33.6°C, the mean being 30.0 ± 2.9°C. The relative humidity ranged from 24.4% to 67.8% with a mean of 46.2 ± 12.9%. The light intensity in the cage ranged from 115 Lx at the bottom to 1988 Lx at the top of the cage. The mean light intensity at the bottom, the tree and the top was 517.2 ± 390.8 Lx, 657.2 ± 280.2 Lx and 542.7 ± 279.2 Lx respectively.

Similar to previous walk-in field cage experiments conducted in South Africa, male and female flies dispersed immediately after release and settled in the more shaded areas in the top of the cage on the black band that connects the ceiling to the vertical panels [43]. Some flies remained immobile after release until recaptured and did not mate. After release, there was usually an immediate mating response, with an overall minimum mating latency time of 2 minutes.

#### Mating performance of sterile and fertile males

**Fig 1:**
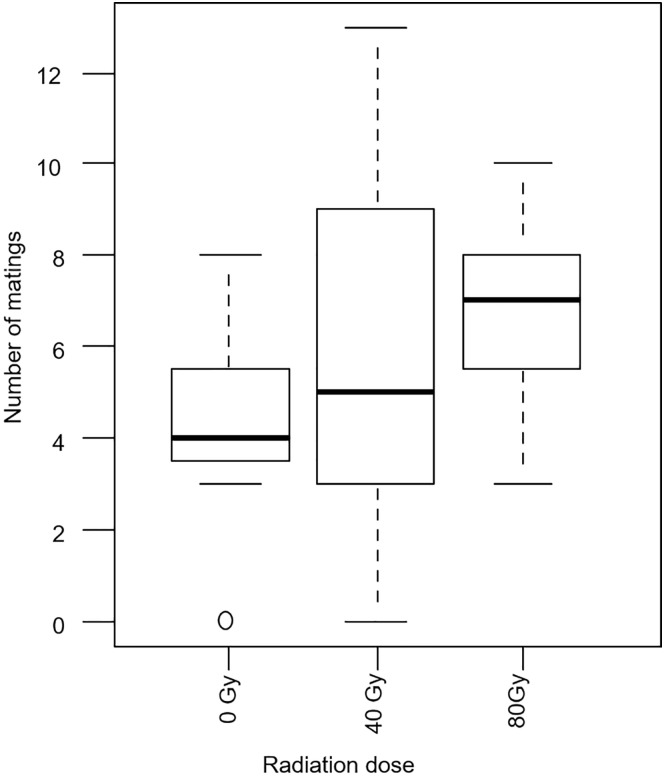
Number of irradiated male (40 or 80 Gy) and untreated male (0 Gy) *Glossina brevipalpis* that mated with untreated females in a field cage. Each box shows the group median separating the 25^th^ and 75^th^ quartiles, capped bars indicate maximum and minimum values, circles indicating the outliners.

The overall propensity of mating (PM) was 0.57 and the RMI obtained for untreated (0 Gy) (0.26 ± 0.12), and 40 Gy (0.33 ± 0.19) and 80 Gy-treated (0.41 ± 0.13) males were not significantly different (P = 0.08) (Fig 1). The RMP of -0.07 of untreated males and males irradiated as adults with 40 Gy indicated that the mating performance of these two groups was almost equal.

Untreated males on average formed mating pairs sooner (mean mating latency 61.1 ± 0.04 min) and on average mated longer (225.1 ± 0.05 min) than the males irradiated with 40 Gy (71.8 ± 0.51 min) (215.8 ± 0.05) and 80 Gy (68.6 ± 0.04 min) (218.6 ± 0.05).

After separation of the mating pairs, the females and males were collected and kept separate in individual fly holding cages for 60 days. As was done for the dose response experiments, female survival and pupae production were monitored and surviving females were dissected after 60 days. More than 80% of all females survived up to day 60 and results on fecundity and abortions were similar to those obtained with the dose response experiments (Tables 2 and 3).

## Discussion

The SIT component of AW-IPM programmes can only be successful if the released sterile males are able to compete with their native counterparts. The selection of the optimal radiation dose to sterilise the insects is important: a dose below the optimal will result in insects that are not adequately sterile and a too high dose can negatively impact on the quality of these insects and may result in insects that are not competitive with wild flies [46–48]. Factors such as the developmental stage, age of the insect and the atmosphere used during irradiation can influence the level of sterility achieved with a specific dose [46,47].

As was previously shown, the proportions of dominant lethal mutations induced in the sperm of *G*. *brevipalpis* increased with increasing radiation dose [31,32], and as a result the rate of induced sterility in mated untreated females likewise increased proportionally with increasing radiation dose. In the present study, mating of males treated as adults with 40 and 80 Gy induced 93% and 99% sterility, respectively in untreated *G*. *brevipalpis* females that mated with the treated males. The induced sterility increased to 97% or more when the males were irradiated with 40 Gy as pupae. These results are in accordance with those of Vreysen et al. [31], who reported that a dose of 50 Gy administered to 4- to 6-day-old males in air induced about 95% sterility in untreated females. The dose required to induce more than 93% dominant lethal mutations in the sperm of *G*. *brevipalpis* is much lower as compared with species such as *Glossina fuscipes fuscipes* Newstead, *G*. *tachinoides* [31], *G*. *pallidipes* [35], *G*. *morsitans* [49] and *G*. *austeni* [50]. Unlike the other species tested, *G*. *brevipalpis* belongs to the fusca group, which have a different chromosome structure than the members of the morsitans or palpalis groups. Although it was suggested that this higher radiation susceptibility might be chromosome related, the main reasons for this still needs to be elucidated [31].

The longer emergence periods and high variation in emergence rate of irradiated pupae obtained in the present study may be an artefact of the basic rearing conditions in the colony. Due to logistical constrains the two species are kept under the same rearing conditions at the ARC-OVI, which may not be the most optimal. Furthermore, due to the variation in emergence rates obtained for the different irradiation doses and time of irradiation, no clear effect of these variables could be detected.

The relatively higher radiation sensitivity of *G*. *brevipalpis* pupae compared to that of adults obtained in the present study are supported by previous studies, i.e. the production rate relative to the untreated control of *G*. *p*. *palpalis* irradiated with 80 Gy was 7.1% and 4.08% when treated as adults and pupae, respectively [34].

Dissection results revealed a clear abortion pattern for female flies that mated with treated males irrespective of life stage, with the rate of abortions increasing with an increase in radiation dose. The uterus of females that mated with irradiated males either contained an egg or was empty due to abortion of the egg in embryonic arrest or an immature larva. It was suggested that these reproductive abnormalities could be used to monitor the impact of sterile male releases on a natural tsetse population [51]. The imbalance between uterus content and the follicle next in ovulation sequence [31] was indeed used to monitor induced sterility in the wild female *G*. *austeni* population in the eradication campaign on Unguja Island, Zanzibar [22]. To assess the rate of induced sterility in the native females of the target population (as a result of a mating with a released sterile male), the natural abortion rate of a population needs to be determined and deducted from the abortion rates observed during a sterile male release programme. Our dissection results clearly indicate that this method of assessing reproductive abnormalities in females in a population under sterile male releases can be used to monitor induced sterility in a targeted *G*. *brevipalpis* population, and hence, as an indicator of programme progress [28]. In addition, the applied radiation doses did not affect *G*. *brevipalpis* males’ insemination ability whether irradiated as adults or as pupae.

The reduction in average longevity of irradiated males is a manifestation of the somatic damage caused by irradiation [22]. Irradiating younger pupae of *Anopheles* (Diptera; Culicidae) mosquitoes and the date moth, *Ectomyelois ceratoniae* (Lepidoptera; Pyralidae) reduced adult survival [52,53] and pupae emergence [54–56] more as compared with irradiating older pupae. This phenomenon was also clearly observed in our study as irradiation as pupae reduced adult longevity more than a treatment as adults.

Data on survival of irradiated insects in the laboratory provides relative information regarding the impact of different doses on male survival as compared with the survival of untreated males. Male survival in the laboratory is most likely not a true reflection of their survival in the field, as the latter will be influenced by many factors other than radiation, and care needs to be taken when extrapolating laboratory survival data to a field situation in an operational sterile male release programme. Although it is important that released males live as long as possible in the wild, the period that they remain sexually active and be able to transfer sperm and outcompete their native counterparts, is the more important aspect. Lifespan and capability of sperm transfer may not be directly related and this requires further research.

Exposure to ionising radiation may affect the biological quality of the released insects [57], and the effect is often dose dependent. With some mosquito species the sterilising dose may be very low (e.g. 35 Gy induced > 95% sterility in *Aedes albopictus*) with minimal impact on male competitiveness (male *Ae*. *albopictus* treated with 35 Gy showed a sexual competitiveness of 0.53 in walk-in field cages five days after emergence) [58]. Lepidoptera on the other hand, have a special chromosome structure with diffuse centromeres, and a much higher dose (250 Gy or more) is usually required to sterilise these males [45].

Our study showed that irradiation doses of up to 80 Gy did not affect the ability of sterilised colony *G*. *brevipalpis* males to compete with fertile colony males for untreated colony females in walk-in field cages. This is in accordance with field cage evaluations that showed that the competitiveness of *G*. *pallidipes* males irradiated with a dose of 120 Gy did not differ from that of untreated ones [37]. In our study, untreated fertile *G*. *brevipalpis* males did form mating pairs sooner and mated for longer than the irradiated males. Although not statistically significant in the present study, any delay in initial mating by irradiated males may potentially reduce their competitiveness in the field. Females that mated with untreated fertile *G*. *brevipalpis* males did have a larger spermathecal fill than those that mated with irradiated males.

The results of the present mating performance studies in walk-in field cages indicated that the colonised *G*. *brevipalpis* seemed to be well suited for use in programmes that have a SIT component. The absence of any noteworthy differences in the quality of flies when irradiated as adults or pupae indicated that *G*. *brevipalpis* can be irradiated either as adults or as late stage pupae, i.e. ≥ 37 days PL. The mating performance of *G*. *brevipalpis* males irradiated as pupae still needs to be compared with that of fertile males.

There are positive and negative aspects to take into consideration in deciding to irradiate adult flies or pupae. Treating pupae has the operational advantage that larger numbers can be irradiated at a time, handling and transport of the immobile pupa are less cumbersome and pupae are less fragile than adult flies. However, the current relative crude sex separation protocol, based on development temperature [59,60] results a certain proportion of females being irradiated and these can therefore not be used for further production in the colony. This is counterproductive because of the limited offspring produced by tsetse flies and all available females are needed.

Our results showed that *G*. *brevipalpis* males were highly sensitive to irradiation and could be sterilised at a much lower dose than some other tsetse fly species. As a result, sterilised colony males proved to be very competitive with un-irradiated colony males for untreated colony females in walk-in field ages. However, the mating performance of irradiated colonised *G*. *brevipalpis* males when competing with fertile wild males under open field conditions still needs to be assessed.
